# Plasma, serum, albumin, and divalent metal ions inhibit the adhesion and the biofilm formation of *Cutibacterium (Propionibacterium) acnes*

**DOI:** 10.3934/microbiol.2018.1.165

**Published:** 2018-03-05

**Authors:** Tatyana V. Polyudova, Daria V. Eroshenko, Vladimir P. Korobov

**Affiliations:** 1 Laboratory of microorganisms' biochemical development, Institute of Ecology and Genetics of Microorganisms, Ural Branch of Russian Academy of Sciences, Perm, Russia; 2 Faculty of Soil Science, Agrochemistry, Ecology and Merchandising, Perm State Agro-Technological University, Perm, Russia; 3 Chemical Engineering Faculty, Perm National Research Polytechnic University, Perm, Russia

**Keywords:** *Cutibacterium (Propionibacterium) acnes*, adhesion, biofilm, plasma, albumin, zinc, hemagglutination

## Abstract

Adhesion and biofilm formation of human skin bacteria *C. acnes* on plasma, serum and albumin-coated polystyrene or in the presence of these blood components were studied. The proteins which were pre-adsorbed to polystyrene surface or added to the medium simultaneously with bacterial cells reduced *C. acnes* adhesion and biofilm formation by 2–5 times to compare to the control. The role of calcium, magnesium and zinc on *C. acnes* attachment was also assessed. Calcium (1 and 10 mM) had the inhibitory effect on *C. acnes* adhesion, whereas zinc (1 and 10 mM) diminished the biofilm formation of *C. acnes*. We also observed that *C. acnes* cells did not bind to erythrocytes. Thus, we suggest that bacteria *C. acnes* preferably colonize the plasma-poor environment due to the inhibitory effect of blood components, in particular, albumin, calcium, and zinc.

## 1. Introduction

Opportunistic bacteria *Cutibacterium* (formerly *Propionibacterium*) *acnes* [1] are a member of the normal human skin and mucous membranes' microbiota [2]. However, *C. acnes* are increasingly recognized as a pathogen, mainly in foreign-body infections, such as arthroplasty or spinal instrumentation-associated infections [3],[4]. The biofilm formation of *C. acnes* are often implicated as a cause of infection following the implantation of joint prostheses, in particular, a shoulder joint [5], intervertebral discs [6],[7], breast prostheses [8] and heart valves [9].

Comparing the places of the most frequent detection of *C. acnes* biofilm, for example, with *S. aureus* which often forms the biofilm on stents, intravenous catheters, central line and etc. [10] it seems that *C. acnes* prefer an environment where the conditions are close to anaerobic and there is no bloodstream. However, only a few studies have provided data on the influence of blood components, especially plasma, on *C. acnes* biofilm formation. Holmberg et al. reported that *C. acnes* primarily infect plasma-poor environments, for example, joint prostheses and cerebrospinal shunts due to an inhibitory action of plasma on the biofilm formation of *C. acnes* [11]. While Bayston et al. claimed that the presence of a plasma conditioning film did not make a significant difference to *C. acnes* adhesion [12]. Thus, the effects of protein-coated surfaces on the adhesion of *C. acnes* remain unclear.

A multitude of proteins are present in human plasma and hence adsorb to the surface of a biomaterial upon blood contact with the formation of so called “conditioning film”. This film plays a major role in determining the interaction of bacteria with biomaterial surfaces. As previous studies have shown this film promotes or inhibits bacterial adhesion. For example, human plasma is responsible for the reduction of *Staphylococcus epidermidis* adhesion and biofilm formation on polyurethane due to the nonspecific interaction and the significant decrease in hydrophobicity [13]. In addition to the conditioning film the plasma proteins are able to interact directly with the surface of bacterial cells [14].

In addition, the influence of plasma on the biofilm formation of pathogenic bacteria is may be also associated with metal ions such as calcium and magnesium, and occasionally zinc [15]–[17]. Moreover, the bacterial capacity to agglutinate erythrocytes as well as biofilm formation is most frequently studied virulence factor [18]. The bacterial ability to directly bind to red blood cells allows them spread in the bloodstream [19]. Unfortunately, the possibility of *C. acnes* to interact with erythrocytes has been poorly studied.

The aim of this study was to examine the role of the plasma, serum and albumin, as well as calcium, magnesium, and zinc in the adhesion mechanism of *C. acnes*.

## 2. Materials and methods

### 2.1. Bacterial strains, cell growth and media

The type bacterial strain *C. acnes* ATCC^®^6919 (received as *P. acnes* VKM Ac-1450 from All-Russian Collection of Microorganisms, Pushchino, Russia) was used in this study. Bacteria from an overnight incubation at 37 °C in Luria-Bertani (LB) broth (10 g tryptone, 5 g yeast extract, 6.4 g KCl and Milli-Q water to 1l; Sigma-Aldrich) were harvested by centrifugation (13,000 rpm, 5 min), washed twice with 0.85% NаCl and diluted to 5 × 10^7^ CFU/ml in LB medium.

Human whole plasma was prepared from blood drawn by venapuncture from three healthy donors. Blood was collected in vacuum tubes with EDTA (Improvacuter, China) and centrifuged (4,000 rpm, 5 min), and the supernatant was diluted in 10 times by 0.85% NaCl or LB medium. To study the effect of simultaneous presence of *C. acnes* cells and blood proteins on adhesion and biofilm formation the LB medium containing either 10% (v/v) plasma, 10% (v/v) heat inactivated fetal bovine serum (Biolot, Russia), or 5 mg/ml bovine serum albumin (Sigma-Aldrich, USA) was used. The medium supplemented with 0.85% NaCl was used as the control.

To examine the role of divalent metal ions on *C. acnes* adhesion and biofilm formation the LB medium supplemented with 0.1, 1 or 10 mM of ZnCl_2_, CaCl_2_ or MgCl_2_ was used. The LB medium was used as the control.

### 2.2. Pretreatment of polystyrene surface by proteins

To investigate the influence of proteins on *C. acnes* adhesion and biofilm formation human plasma (10%), heat inactivated fetal bovine serum (10%) and bovine serum albumin (5 mg/ml) were pre-adsorbed to the polystyrene surface for 24 h prior to the bacterial adhesion or the biofilm formation assay. The control group for protein-coated surfaces was incubated in 0.85% NaCl without proteins.

### 2.3. Adhesion assay

Bacterial adhesion was studied in static conditions in protein-coated or untreated 40 mm polystyrene Petri dishes. For this, *C. acnes* cells (2 ml/dish) allowed adhering at 37 °C for 60 min. After the triple rinsing with PBS and the staining with 0.1% crystal violet the mean of the number of the adherent bacterial cells was counted at least in 10 field-of-view (FOV) for each dish using the digital optical microscope (µViso-103; Lomo, Russia) at magnification ×1000.

### 2.4. Biofilm formation assay

The biofilm formation of *C. acnes* was carried out according to the standard procedure in a 96-well flat-bottom polystyrene plate [20] with or without protein-coating (Medpolimer, Russia) in appropriate medium at 37 °C for 48 h. The biomass of the biofilm was determined as OD_570_ of the ethanol extract after dyeing with 0.1% crystal violet for 20 min using a microtiter plate reader (Benchmark PlusMicroplate Spectrophotometer System, Bio-Rad, USA).

### 2.5. Adhesion of C. acnes to erythrocytes

The level of the adhesion of *C. acnes* to erythrocytes was accessed according to the method of Rupp & Archer [21]. Briefly, fresh human erythrocytes (blood type O, Rh+) were washed twice in PBS and diluted to 1% by PBS. Beforehand a series of two fold dilutions of *C. acnes* suspension starting from 10^8^ CFU/ml in the 96-well round bottom plate was prepared. Then, the bacteria were mixed with equal amounts of erythrocytes (100 µl/well) and incubated for 2 h at room temperature. The level of hemagglutination was judged by the formation of a precipitate of erythrocytes in the well. The number of the adherent to erythrocytes bacterial cells was counted after dyeing with 0.1% crystal violet for 10 min at least for 100 erythrocytes using the digital optical microscope (µViso-103; Lomo, Russia) at magnification ×1000.

### 2.6. Statistics

Statistical analysis of the data was carried out using the ANOVA method. The results were presented as M ± SD for three independent experiments. The differences at *p* < 0.05 were considered reliable.

## 3. Results and discussion

### 3.1. Effect of plasma, serum, and albumin on adhesion and biofilm formation of C. acnes

**Figure 1.**
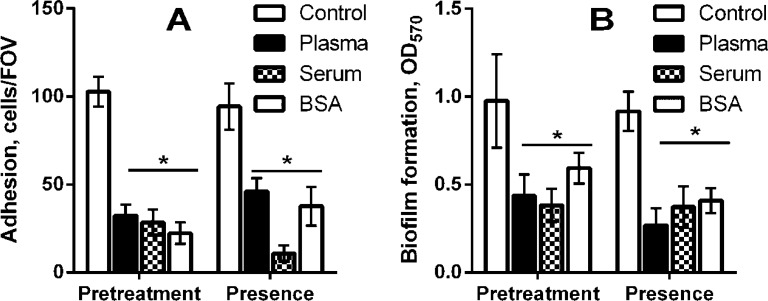
Adhesion (A) and biofilm formation (B) of *C. acnes* ATCC 6919 on polystyrene surface coated by or in the presence of 10% plasma, 10% serum and 0.5% albumin. The error bars indicate the standard deviations of the means for three experiments.

Bacterial adhesion and biofilm formation of *C. acnes* ATCC 6919 on protein-coated and untreated polystyrene surfaces are shown in Figure 1. The bacterial adhesion significantly decreased when surface was coated with plasma, serum or albumin compared to untreated polystyrene (Figure 1A). The similar effect was observed when proteins were added to the medium simultaneously with bacterial cells. It is interesting to note that serum caused almost twice more pronounced inhibitory effect compared with plasma or albumin.

The inhibitory effect of blood proteins against *C. acnes* ATCC 6919 continued even after incubation for 48 h. As shown in Figure 1B the biomass of 2-day old biofilm of *C. acnes* ATCC 6919 on the protein-coated polystyrene was lower than control by 30%–60%. A similar situation was observed in the presence of proteins. So, *C. acnes* biofilm formation was about twice slower in LB medium supplemented with plasma, serum and albumin to compare the control (Figure 1B).

Thus, the protein coating of the polystyrene surfaces reduced the amount of bacterial adhesion as well as the biofilm formation of *C. acnes*. Moreover, the attachment and biofilm formation of *C. acnes* in the presence of plasma, serum and albumin was quite also low. Hence, our results which show the significant influence of blood components on the *C. acnes* adhesion and biofilm formation are opposing to the results of Bayston et al. [12], but support the hypothesis of Holmberg et al. [11]. The mechanism of the inhibition of the bacterial attachment to plasma or albumin-coated polymeric surface is often related to the decrease in the surface hydrophobicity [13]. Whereas antibiofilm activity of human serum against S. epidermidis is associated with apo-Transferrin [22]. Although apo-Transferrin was found to suppress *S. epidermidis* biofilm production, it had no effect on *S. epidermidis* initial adhesion. Therefore, the inhibitory effect of serum on the first stages of biofilm formation is probably also mediated by albumin.

Additionally, *C. acnes* have 80 kDa surface-associated, fibronectin-binding protein [14], which could promote the bacterial adhesion on the plasma-coated surface, but in our experiments, we observed the opposite effect (Figure 1A). This effect may depend on the bacterial strain probably due to differences in bacterial cell surface properties.

### 3.2. Effect of calcium, magnesium, and zinc on adhesion and biofilm formation of C. acnes

However, as shown in earlier studies the bivalent metal ions, such as calcium and magnesium, also control the adhesive properties of bacterial cells, for example, staphylococci [15],[16]. Therefore, we investigated the effect of Ca^2+^ and Mg^2+^ ions on the adhesion and biofilm formation of *C. acnes* in concentrations similar to their level in the serum (∼1 mM) and in 10 times lower and higher (0.1 mM and 10 mM, respectively). Despite the low concentration of zinc in the blood (0.1–0.2 mM) we also evaluated the effect of Zn^2+^ on the adhesion and biofilm formation of *C. acnes* due to the widespread use of zinc-containing drugs for the treatment of acne [23].

**Figure 2.**
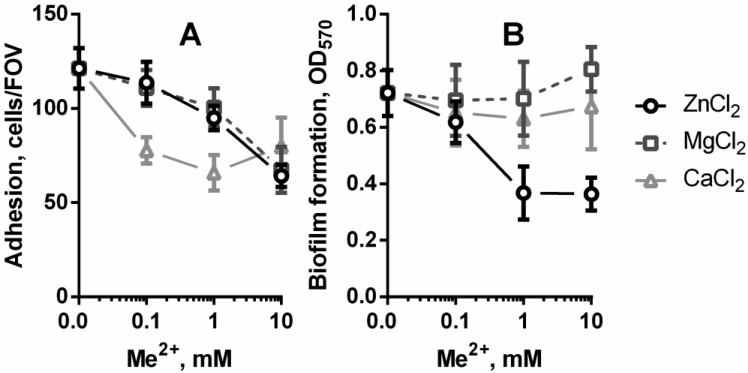
Adhesion (A) and biofilm formation (B) of *C. acnes* ATCC 6919 on polystyrene surface in LB medium supplemented with 0.1, 1 and 10 mM of CaCl_2_, MgCl_2_ or ZnCl_2_.

Bacteria *C. acnes* exhibited different abilities to adhere and form biofilm in the presence of divalent ions as shown in Figure 2. Calcium inhibited the adhesion of *C. acnes* ATCC 6919 to polystyrene approximately by 1.5 times in all tested concentrations (0.1, 1.0 and 10 mM). While the presence of 0.1 and 1 mM Mg^2+^ and Zn^2+^cations in the LB medium did not result in the significant inhibition of the adhesion of bacterial cells. The presence of Mg^2+^ and Zn^2+^ at concentrations in 10 and 100 times, respectively, higher than their serum levels caused almost 2 fold decrease in the number of attached cells compared to the control. The elongation of the incubation to 48 h resulted in a bit different result outcome, so the biomass of *C. acnes* biofilm was significantly lower than control only in the medium supplemented with zinc ions (Figure 2B). Moreover, the inhibitory effect of 1 mM and 10 mM Zn^2+^ was similar and the biofilm formation was diminished by 50%. In contrast to zinc, magnesium and calcium ions did not appear to have any effect on the biofilm formation of *C. acnes*.

Thus, we found that calcium at the concentration in the range of normal level in the blood (1.05–1.23 mM) had the greatest effect on the first stages of biofilm formation. We also observed the small deceleration in *C. acnes* adhesion in medium containing 1 mM Mg^2+^. Most likely, divalent cations, such as Mg^2+^ and Ca^2+^, can influence adhesion and biofilm formation directly via non-specific electro-static interactions [24] and indirectly via physiology-dependent attachment processes by acting as important cellular cations and enzyme cofactors [15],[16]. The presence of Zn^2+^ at non-bactericidal concentrations in growth media can stimulate the *S. aureus* adhesion to abiotic surfaces via the activation of cell-cell adhesion [25], and vice versa, Zn^2+^ caused the decrease of adhesive properties of *Streptococcus suis* [17]. Unfortunately, the role of zinc in the adhesion and biofilm formation of *C. acnes* has been poorly studied, but Ozuguz et al. [26] have found that the plasma levels of zinc in all patients with acne vulgaris were decreased almost in two times and this finding correlates with acne severity. Thus, the decrease of zinc concentration in blood plasma can lead to the development of *C. acnes* biofilm. Furthermore, in the light of these data we suggest that the effect of zinc-containing drugs used for the prevention and treatment of acne can be mediated not only by the regulation of the activity of sebaceous glands via the testosterone production [27], but also bound with the inhibitory activity of zinc against *C. acnes* adhesion and biofilm formation.

### 3.3. Adhesion of C. acnes to erythrocytes

**Figure 3.**
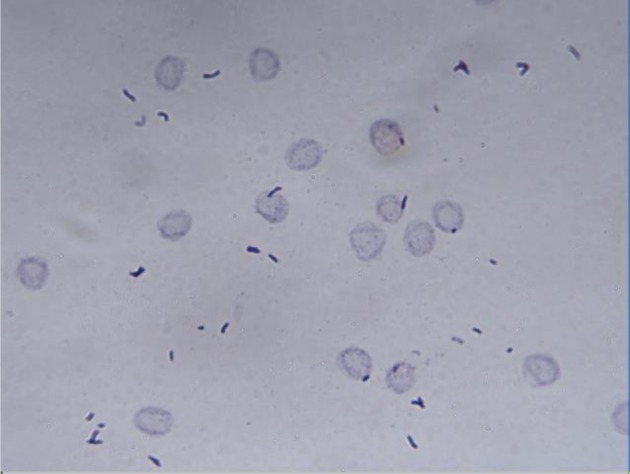
The absence of the adhesion of *C. acnes* ATCC 6919 to human erythrocytes (O, Rh+) after 2 h of the incubation (magnification ×1000).

Hemagglutination of erythrocytes by microbes was often used as a marker of bacterial pathogenicity [18]. There was found that the ability of *S. epidermidis* to hemagglutinate erythrocytes correlated with the bacterial adherence to intravenous catheters [21]. In relation to *C. acnes* ATCC 6919, only their ability to internalize in osteoblasts and osteoclasts and survive intracellularly for at least 96 h has recently been shown [28]. Therefore, we evaluated the possibility of binding *C. acnes* cells with human erythrocytes. In our experiments we observed the absence of binding of *C. acnes* ATCC 6919 cells with erythrocytes (Figure 3) or the formation of a precipitate in the well similar to dairy propionibacteria [29]. The fact that *C. acnes* did not bind to erythrocytes may be the additional evidence of the assumption about the priority colonization of blood-poor environments by *C. acnes*.

## 4. Conclusions

The results of our studies indicate the high sensitivity of the first stages of the *C. acnes* biofilm formation to the blood components. So, the protein-coated surface (albumin, plasma) that could be formed on the prostheses in human body becomes unfavorable for the adherence and the biofilm formation of *C. acnes*. In addition, calcium and zinc can cause the decrease of *C. acnes* attachment. Therefore, the bacterial adhesion on the polystyrene depends on proteins and cations presenting in human blood. Thus, the results of this study confirm that bacteria *C. acnes* preferably colonize the plasma-poor environment due to the inhibitory effect of albumin, calcium and zinc.
